# Incretin-Based Therapies, Obesity-Associated Inflammation, and Atherosclerotic Cardiovascular Risk

**DOI:** 10.3390/cells15141293

**Published:** 2026-07-20

**Authors:** Jan Kafol, Borut Jug, Zlatko Fras

**Affiliations:** 1 Department of Vascular Diseases, Division of Internal Medicine, University Medical Centre Ljubljana, 1000 Ljubljana, Slovenia; 2 Faculty of Medicine, University of Ljubljana, 1000 Ljubljana, Slovenia

**Keywords:** atherosclerosis, inflammation, residual inflammatory risk, obesity, glucagon-like peptide-1 receptor agonists, semaglutide, tirzepatide, incretin-based therapy, cardiovascular prevention, adipose tissue inflammation

## Abstract

Cardiovascular disease remains a leading cause of mortality despite major advances in lipid lowering and risk-factor control, highlighting the importance of residual cardiovascular risk. Inflammation is a central driver of atherosclerosis, while obesity promotes chronic low-grade inflammation, adipose tissue dysfunction, ectopic fat accumulation, and vascular injury. This narrative review focuses on obesity-associated inflammation as an upstream contributor to residual atherosclerotic risk and evaluates whether incretin-based therapies modify this pathway through weight loss, metabolic improvement, and additional inflammatory or vascular mechanisms. Data from mechanistic studies, biomarker analyses, vascular imaging studies, and cardiovascular outcome trials are reviewed. Anti-inflammatory trials support inflammation as a modifiable therapeutic pathway, although clinical benefit depends on the therapeutic target, timing, and patient selection. Glucagon-like peptide-1 receptor agonists reduce inflammatory and oxidative stress biomarkers and show anti-atherosclerotic effects in experimental models, but human vascular imaging data remain inconclusive. Cardiovascular outcome trials establish benefit with several GLP-1 receptor agonists, including semaglutide in selected patients with overweight or obesity without diabetes. However, direct human evidence for receptor-mediated anti-inflammatory or anti-atherosclerotic effects remains limited, and the relative contributions of weight loss, metabolic improvement, and additional mechanisms remain uncertain.

## 1. Introduction

Over the past several decades, advances in cardiovascular prevention and treatment have substantially reduced cardiovascular morbidity and mortality in many developed countries. Nevertheless, cardiovascular disease remains the leading cause of death worldwide [1,2]. While optimization of traditional cardiovascular risk factors has contributed substantially to reductions in coronary mortality [3], major modifiable risk factors account for approximately half of incident cardiovascular disease [4]. This observation highlights the existence of substantial residual cardiovascular risk and has stimulated the search for additional therapeutic targets. Among the mechanisms contributing to residual cardiovascular risk, inflammation has emerged as a fundamental driver of atherosclerosis and an increasingly important therapeutic target [5,6,7].

The concept of atherosclerosis as a chronic inflammatory disease has advanced considerably, shifting from a lipid-centric model toward a more integrated view incorporating immune and inflammatory pathways [8]. While retention and modification of apolipoprotein B-containing lipoproteins within the arterial wall remain central drivers of atherogenesis, inflammatory processes modulate virtually every stage of disease development and progression [5,9]. Endothelial dysfunction promotes the recruitment of circulating monocytes and other immune cells into the vascular intima, where they contribute to foam cell formation and the development of atherosclerotic plaques [10,11]. As lesions progress, sustained activation of innate and adaptive immune pathways drives plaque growth, vascular remodeling, and ultimately plaque destabilization, increasing the risk of thrombotic cardiovascular events [10,11].

The clinical relevance of inflammation in atherosclerosis extends beyond mechanistic observations. Accumulating evidence has demonstrated that inflammatory biomarkers, particularly high-sensitivity C-reactive protein (hsCRP) and interleukin-6 (IL-6), predict cardiovascular events independently of traditional risk factors and lipid concentrations [12]. Furthermore, while intensive lipid-lowering therapy substantially reduces cardiovascular risk, many patients continue to experience recurrent cardiovascular events despite achieving recommended low-density lipoprotein cholesterol (LDL-C) targets [13]. This observation has led to the concept of residual inflammatory risk and prompted investigations into whether direct modulation of inflammatory pathways could further improve cardiovascular outcomes [14]. Clinical trials of interleukin-1β (IL-1β) inhibition and low-dose colchicine have established inflammation as a modifiable component of atherosclerotic risk, although benefit appears to depend on the targeted pathway, clinical context, and treatment safety [7,15,16].

Among the conditions contributing to residual inflammatory risk, obesity has emerged as one of the most prevalent and clinically important. Once considered merely a disorder of excess energy storage, obesity is now recognized as a state of chronic low-grade systemic inflammation characterized by adipose tissue dysfunction, immune cell infiltration, and increased production of pro-inflammatory mediators, including tumor necrosis factor-α (TNF-α), IL-6, and C-reactive protein (CRP) [17,18]. These inflammatory alterations not only contribute to metabolic dysfunction but also promote endothelial dysfunction, vascular inflammation, and accelerated atherosclerosis, providing a mechanistic link between obesity and cardiovascular disease [19]. Importantly, obesity-associated inflammation may represent an upstream driver of residual inflammatory risk, suggesting that interventions targeting obesity could confer cardiovascular benefit through weight loss, metabolic improvement, and additional mechanisms affecting inflammation and vascular biology, although clearly weight-independent effects remain unproven [12,17,19].

In this context, incretin-based therapies have become important cardiometabolic agents. Originally developed for the treatment of type 2 diabetes and subsequently approved for obesity management, several glucagon-like peptide-1 receptor agonists (GLP-1RAs) have reduced cardiovascular events in large outcome trials, whereas the dual glucose-dependent insulinotropic polypeptide (GIP)/glucagon-like peptide-1 (GLP-1) receptor agonist tirzepatide demonstrated noninferiority, but not superiority, to dulaglutide for major adverse cardiovascular events [20,21]. However, the relative contributions of weight loss, metabolic improvement, and additional inflammatory or vascular mechanisms to these cardiovascular benefits remain uncertain [22,23].

**Figure 1:**
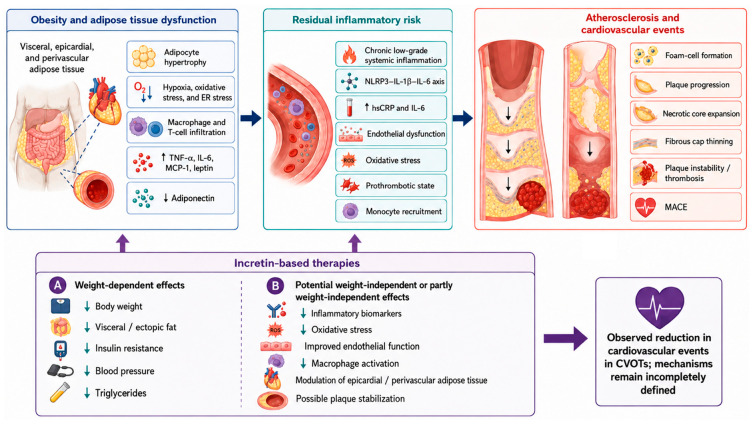
Incretin-based therapies, obesity-associated inflammation, and atherosclerosis. Obesity promotes adipose tissue dysfunction, chronic low-grade inflammation, endothelial dysfunction, and plaque progression, thereby contributing to residual inflammatory risk and cardiovascular events. Incretin-based therapies may reduce cardiovascular risk through weight loss, improvements in cardiometabolic risk factors, and other potential mechanisms involving inflammation, oxidative stress, endothelial function, macrophage activation, and ectopic adipose tissue biology. **Abbreviations**: CVOT, cardiovascular outcome trial; ER, endoplasmic reticulum; hsCRP, high-sensitivity C-reactive protein; IL, interleukin; MACE, major adverse cardiovascular events; MCP-1, monocyte chemoattractant protein-1; NLRP3, NOD-like receptor family pyrin domain-containing 3; ROS, reactive oxygen species; TNF-α, tumor necrosis factor-α.

This review addresses a focused question: how obesity-associated inflammation contributes to residual atherosclerotic risk and how incretin-based therapies may modify this pathway. We therefore examine adipose and vascular mechanisms, receptor biology, human biomarker and imaging data, and cardiovascular outcome evidence, while distinguishing established clinical benefit from still-unproven weight-independent anti-inflammatory or vascular mechanisms. The proposed pathway is summarized in Figure 1.

## 2. Literature Search and Study Selection

This narrative review was informed by a targeted search of PubMed, supplemented by Google Scholar, Scopus, major cardiovascular and diabetes journals, and reference-list screening, from database inception to 12 July 2026. Search terms included combinations of “atherosclerosis,” “vascular inflammation,” “residual inflammatory risk,” “obesity,” “adipose tissue inflammation,” “NLRP3,” “GLP-1 receptor agonist,” “GIP,” “tirzepatide,” “semaglutide,” “endothelial dysfunction,” “macrophage,” “foam cell,” “vascular imaging,” and “cardiovascular outcomes.” Priority was given to randomized cardiovascular outcome trials, systematic reviews and meta-analyses, human biomarker and imaging studies, major guidelines, and mechanistic studies directly relevant to obesity-associated inflammation and incretin-based therapy. Preclinical studies were included when they provided otherwise unavailable information on adipose, immune, endothelial, or plaque mechanisms. Older landmark studies were retained where necessary, whereas studies focused solely on glycemic efficacy were not emphasized. Articles were selected according to relevance, methodological quality, and contribution to the review question; no formal systematic-review protocol or meta-analysis was performed.

## 3. Inflammation in Atherosclerosis

Although atherosclerosis was historically viewed primarily as a disorder of lipid accumulation, it is now recognized as a chronic inflammatory disease of the arterial wall [8,10,24]. The retention and modification of apolipoprotein B-containing lipoproteins within the arterial intima initiate a complex interplay between lipid deposition and immune activation that drives all stages of atherogenesis. Modified low-density lipoprotein (LDL) particles trigger endothelial activation and dysfunction, promoting the recruitment of circulating monocytes and other immune cells into the vessel wall. These cells differentiate into macrophages, internalize modified lipoproteins, and form foam cells, contributing to the development of early atherosclerotic lesions. As plaques progress, persistent activation of innate and adaptive immune pathways promotes vascular remodeling, necrotic core formation, fibrous cap weakening, and ultimately plaque rupture and thrombosis [9,10,11,25].

Among the numerous inflammatory pathways implicated in atherosclerosis, the NOD-like receptor family pyrin domain-containing 3 (NLRP3) inflammasome–IL-1β–IL-6 signaling axis has emerged as particularly important. Cholesterol crystals and oxidized lipoproteins activate the NLRP3 inflammasome within macrophages, resulting in the production of IL-1β [26,27]. IL-1β subsequently stimulates downstream release of IL-6, amplifying local vascular inflammation and promoting systemic inflammatory responses. One consequence of IL-6 signaling is increased hepatic synthesis of CRP, which has become the most widely used clinical biomarker of residual inflammatory risk [28].

The importance of the NLRP3–IL-1β–IL-6 pathway is supported by extensive epidemiological and clinical evidence demonstrating that elevated concentrations of IL-6 and hsCRP predict future cardiovascular events independently of lipid concentrations. Furthermore, residual inflammatory risk and residual cholesterol risk appear to represent partially independent contributors to cardiovascular events, highlighting the multifaceted nature of atherosclerotic disease [12,13,29]. These observations have established inflammation as a central component of atherogenesis and provided the biological rationale for the development of therapies specifically targeting inflammatory pathways in cardiovascular disease [7,15,16].

## 4. Targeting Inflammation in Atherosclerotic Cardiovascular Disease

Statins provided early evidence that reduction in vascular inflammation may contribute to cardiovascular benefit. In addition to lowering LDL-C, statins reduce hsCRP and exert pleiotropic effects on endothelial function, oxidative stress, leukocyte recruitment, and nuclear factor kappa B (NF-κB) signaling [6,30]. In JUPITER, rosuvastatin reduced cardiovascular events in individuals with elevated hsCRP despite relatively low LDL-C, although the trial could not separate the effects of lipid lowering from inflammation reduction [31].

The CANTOS trial provided direct proof-of-concept that inflammation is a modifiable therapeutic target in atherosclerosis. In patients with previous myocardial infarction and persistent hsCRP elevation, selective inhibition of IL-1β with canakinumab reduced recurrent cardiovascular events without lowering LDL-C [7]. Benefit was greatest among patients with larger reductions in IL-6 and hsCRP, supporting the importance of the NLRP3–IL-1β–IL-6 pathway [28]. However, the overall benefit was moderate, and fatal infections were more frequent, highlighting the need to balance suppression of vascular inflammation against preservation of host defense [7]. Phase 2 treatment with the IL-6 ligand inhibitor ziltivekimab substantially reduced inflammatory and thrombotic biomarkers, but definitive cardiovascular outcome evidence is still required [32].

Low-dose colchicine offers a broader and more pragmatic anti-inflammatory approach. COLCOT and LoDoCo2 demonstrated reductions in cardiovascular events after myocardial infarction and in chronic coronary disease, respectively [15,16]. However, results have not been uniform across clinical settings: the larger CLEAR trial was neutral after acute myocardial infarction [33], and pooled evidence in patients with recent myocardial infarction remains inconclusive [34]. These findings suggest that the efficacy of anti-inflammatory treatment depends on disease stage, timing, patient selection, adherence, and the inflammatory pathway being targeted.

Several other anti-inflammatory strategies have failed to improve cardiovascular outcomes. In the Cardiovascular Inflammation Reduction Trial (CIRT), low-dose methotrexate did not reduce IL-1β, IL-6, hsCRP, or cardiovascular events [35]. Inhibition of lipoprotein-associated phospholipase A2, secretory phospholipase A2, and p38 mitogen-activated protein kinase (MAPK) was also neutral or potentially harmful [36,37,38,39]. Collectively, these trials indicate that nonspecific suppression of inflammation is insufficient and that successful therapy likely requires targeting biologically relevant pathways in appropriately selected patients.

This evidence is particularly relevant to obesity, in which dysfunctional adipose tissue provides a persistent upstream source of inflammatory signaling, including NLRP3 activation, IL-1β, IL-6, and chemokine production [17,40,41,42]. Incretin-based therapies differ from dedicated anti-inflammatory drugs because they primarily reduce adiposity and metabolic dysfunction while potentially also influencing inflammatory, endothelial, and immune pathways [43,44]. Whether these additional effects contribute independently to cardiovascular protection remains uncertain, but obesity-associated inflammation provides a biological rationale for evaluating incretin-based therapy as an upstream strategy for reducing residual inflammatory risk [22,44].

## 5. Obesity as a Driver of Inflammation

Obesity is increasingly recognized not only as excess fat accumulation, but as a state of adipose tissue dysfunction and chronic low-grade inflammation [17,45]. This is particularly evident in visceral and ectopic adipose depots, which are more lipolytically active and more pro-inflammatory than gluteofemoral subcutaneous fat [19,41]. With progressive adipocyte hypertrophy, adipose tissue becomes hypoxic and metabolically stressed, leading to increased free fatty acid release, mitochondrial and endoplasmic reticulum stress, oxidative stress, and activation of innate immune pathways [17,41]. These changes promote insulin resistance, dyslipidemia, endothelial dysfunction, and systemic inflammation, thereby linking obesity to atherosclerotic cardiovascular disease [19,41].

A key feature of this process is altered adipokine secretion. In obesity, the balance shifts from anti-inflammatory and vasoprotective mediators, such as adiponectin, toward pro-inflammatory adipokines and cytokines, including leptin, resistin, TNF-α, IL-6, and monocyte chemoattractant protein-1 (MCP-1) [46,47]. These mediators promote endothelial activation, leukocyte adhesion, monocyte recruitment, vascular oxidative stress, impaired nitric oxide bioavailability, and a prothrombotic state [41,48]. At the same time, macrophage infiltration into adipose tissue sustains local inflammation. In lean, metabolically healthy adipose tissue—that is, adipose tissue in the non-obese state—resident immune cells generally support tissue homeostasis, whereas obesity favors the accumulation of pro-inflammatory macrophages, CD8-positive T cells, T helper type 1 (Th1) cells, neutrophils, and mast cells, creating a self-amplifying inflammatory loop between adipocytes and immune cells [40,49,50,51].

These inflammatory alterations have direct relevance for atherosclerosis. Adipose-derived cytokines and chemokines promote monocyte recruitment into the arterial wall, foam cell formation, smooth muscle cell activation, extracellular matrix remodeling, and plaque vulnerability [41,48,52]. Increased free fatty acid flux and insulin resistance further contribute to hepatic overproduction of apolipoprotein-B-containing lipoproteins and endothelial injury [19,41]. Visceral, epicardial, and perivascular adipose tissue may also influence vascular inflammation through local paracrine signaling [40,53]. Thus, obesity can be viewed as an upstream driver of residual inflammatory risk, providing a rationale for therapies that target obesity while also potentially attenuating vascular inflammation and plaque progression. This concept is particularly relevant for incretin-based anti-obesity therapies, whose cardiovascular benefits may reflect a combination of weight loss, metabolic improvement, and additional effects on inflammation and vascular biology.

## 6. Incretin Physiology, Receptor Distribution, and Cellular Mechanisms

GLP-1 and GIP are nutrient-responsive incretin hormones secreted predominantly by enteroendocrine L cells of the distal small intestine and colon and K cells of the proximal small intestine, respectively. Both hormones are released after nutrient ingestion and are rapidly inactivated by dipeptidyl peptidase-4. The GLP-1 receptor (GLP-1R) and GIP receptor (GIPR) belong to the class B family of G-protein-coupled receptors and signal predominantly through the stimulatory G-protein α-subunit (Gα_s_), which activates adenylyl cyclase and increases cyclic adenosine monophosphate (cAMP), with downstream activation of protein kinase A and exchange protein directly activated by cAMP 2 (EPAC2). In pancreatic β cells, these pathways increase intracellular calcium and potentiate glucose-dependent insulin-granule exocytosis [54,55,56].

Although their pancreatic actions overlap, the physiological profiles of the two incretins are not identical. GLP-1 suppresses glucagon secretion during hyperglycemia, delays gastric emptying, and reduces food intake through vagal, brainstem, hypothalamic, and reward-related neural circuits. GIP similarly potentiates glucose-dependent insulin secretion but can support glucagon secretion at lower glucose concentrations and participates in central regulation of energy balance and peripheral nutrient handling, particularly in adipose tissue [56,57]. Long-acting GLP-1RAs and dual GIP/GLP-1R agonists produce sustained pharmacological receptor activation that differs from the brief postprandial exposure to endogenous incretins; consequently, the actions of agents such as tirzepatide cannot be understood as a simple summation of the physiological effects of native GLP-1 and GIP [57,58].

Beyond the pancreatic islets, GLP-1R and GIPR are expressed in selected neuronal and peripheral compartments, including gastrointestinal, cardiovascular, and adipose tissues, although their distribution is highly cell-, tissue-, and species-specific [44,55,56,57,59,60]. The principal sites of receptor expression and their functional relevance are summarized in Table 1. The evidence supporting individual sites of expression is not equally robust. In particular, GLP-1R is generally expressed at low abundance, and several antibodies used in earlier immunohistochemical studies lacked adequate specificity. Greater weight should therefore be given to findings supported by validated antibodies, ligand-binding studies, genetic reporter models, or transcriptomic approaches [60,61].

Incretin-based therapies may attenuate obesity-associated inflammation through a combination of systemic metabolic effects and tissue-specific receptor-linked mechanisms. By reducing energy intake, body weight, visceral and ectopic adiposity, and insulin resistance, these therapies may attenuate adipocyte hypertrophy, hypoxia, mitochondrial and endoplasmic-reticulum stress, excessive lipolysis, and free-fatty-acid flux. These changes may reduce adipocyte production of C–C motif chemokine ligand 2, also known as MCP-1, TNF-α, and IL-6 and may restore a more favorable adipokine profile, including increased adiponectin [17,40,43]. Because GLP-1R expression in conventional adipocytes is low or inconsistent, these effects are likely to be predominantly indirect for GLP-1RAs [44,55]. GIPR expression in adipose tissue provides a more plausible basis for direct adipose actions of dual agonists, although the specific contribution of adipocyte GIPR signaling to the anti-inflammatory effects of tirzepatide remains unresolved [56,57].

Reduced adipocyte stress and chemokine production may, in turn, limit monocyte recruitment and inflammatory activation within adipose tissue [17,40,43]. Across experimental metabolic and atherosclerosis models, GLP-1R agonism has been associated with lower macrophage inflammatory activity and reduced expression of inflammatory markers, including CD68, TNF-α, IL-1β, and IL-6 [62,63,64]. These changes should not be described simply as an “M1-to-M2 switch,” because adipose tissue macrophages occupy multiple metabolically and transcriptionally distinct states—including lipid-associated and metabolically activated populations—that are not adequately represented by a binary polarization model [65,66,67]. Although direct GLP-1R-linked effects have been reported in human macrophages [68], the importance of macrophage-autonomous GLP-1R signaling in vivo remains uncertain [44,69]. Central GLP-1R activation can suppress peripheral inflammatory responses through adrenergic and opioid pathways, indicating that reduced macrophage inflammation does not necessarily require direct receptor activation in myeloid cells [69].

The NLRP3 inflammasome provides a mechanistic link between adipose dysfunction and vascular inflammation. Lipotoxic and mitochondrial stress in expanding adipose tissue, together with cholesterol crystals and oxidized lipoproteins within atherosclerotic plaques, promote priming and activation of the NLRP3 inflammasome in adipose-tissue and plaque macrophages, leading to caspase-1-dependent maturation and release of IL-1β and interleukin-18 (IL-18) [26,42]. Preclinical evidence suggests that GLP-1R agonism may attenuate this pathway by reducing metabolic and mitochondrial stress, reactive oxygen species, NF-κB signaling, and inflammasome activation [44,62,63].

At the vascular interface, GLP-1RAs have reduced oxidative stress, NF-κB activation, adhesion-molecule expression, and chemokine signaling in experimental models, while increasing endothelial nitric-oxide synthase activity and nitric-oxide bioavailability. These changes may reduce monocyte adhesion and transendothelial recruitment, thereby interrupting an early step in atherogenesis [63,70,71]. Endothelial GLP-1R-dependent protection has been demonstrated in genetically modified mouse models [72], but reproducible receptor expression in human vascular endothelial cells remains uncertain; therefore, direct endothelial signaling and indirect metabolic, neural, or paracrine mechanisms remain plausible [44,60]. Using a validated monoclonal antibody and ligand-binding methods, Pyke et al. did not detect GLP-1R in endothelial cells within the assessed primate vascular compartments [60].

Within the arterial wall, GLP-1R agonism has been associated with reduced uptake of modified lipoproteins, attenuation of macrophage foam-cell formation, and decreased plaque macrophage content and inflammatory gene expression [63,64,73]. Liraglutide and semaglutide reduced lesion development in mice deficient in apolipoprotein E (ApoE) or the LDL receptor, accompanied by reductions in leukocyte recruitment and plaque inflammatory pathways [64]. More recently, liraglutide reduced plaque progression, macrophage content, cathepsin activity, and systemic inflammatory biomarkers in normoglycemic, non-obese rabbits [23]. These findings support effects on plaque macrophage accumulation and function but do not establish direct GLP-1R signaling in human plaque macrophages [44,60].

Consistent with these experimental mechanisms, a systematic review and meta-analysis of 40 randomized trials found that GLP-1RAs reduced CRP, TNF-α, and malondialdehyde and increased adiponectin, whereas effects on IL-6 were less consistent [43]. However, changes in circulating biomarkers cannot establish the responsible cell type or distinguish direct receptor-mediated effects from those secondary to weight loss and metabolic improvement [43,44]. For tirzepatide, direct vascular evidence remains preclinical: treatment reduced aortic MCP-1, IL-6, intercellular adhesion molecule-1 (ICAM-1), and CD68 expression in ApoE-deficient mice, but the specific contribution of GIPR activation remains unresolved [44,57,74].

**Table 1:** Current evidence for the tissue and cellular distribution of GLP-1R and GIPR and the potential functional relevance of receptor activation.

Tissue or Cellular Compartment	GLP-1R Distribution	GIPR Distribution	Potential Functional Relevance
Pancreatic islets	Predominantly expressed in β cells; lower expression has been reported in δ cells, whereas α-cell expression is limited and species-dependent [54,55,56,60]	Prominent in β cells and also detected in α cells; distribution among other islet-cell populations is species-dependent [56,57]	Glucose-dependent insulin secretion and paracrine regulation of glucagon and somatostatin secretion
Central and autonomic nervous systems	Discrete hypothalamic, brainstem, reward-related, and autonomic neuronal populations [55,56]	Hypothalamic and other neuronal populations involved in energy balance; detailed cellular mapping remains predominantly preclinical [57,59]	Regulation of appetite, food reward, energy balance, autonomic output, and neuroimmune signaling
Gastrointestinal tract	Myenteric neurons, selected gastric cells, duodenal Brunner glands, and gastrointestinal smooth muscle; vagal-afferent expression is supported mainly by animal studies [54,55,56,60]	Less comprehensively characterized; cell-specific gastrointestinal distribution remains uncertain [56,57]	GLP-1R-mediated regulation of gastric emptying, gastrointestinal motility, and gut–brain communication; direct gastrointestinal GIPR functions are less clearly defined
Cardiovascular system	Demonstrated in sinoatrial-node myocytes and selected vascular smooth-muscle compartments, including renal and pulmonary arteries and arterioles in primates; reproducible human endothelial-cell expression remains uncertain [44,60,72]	Expression has been reported in cardiac and vascular tissues, but cell-specific localization and functional importance in humans remain incompletely established [44,57]	Regulation of heart rate, autonomic cardiovascular function, and vascular tone; direct endothelial and arterial-wall effects in humans remain unproven
Adipose tissue	Low or variable expression in conventional white-adipose depots; tissue-level expression has been reported in human epicardial adipose tissue [44,56,75]	More consistently detected in adipose tissue, although localization varies among adipocytes, stromal-vascular cells, and adipose depots [56,57,76]	Lipid handling, nutrient partitioning, and potential local modulation of adipose-tissue inflammation; the importance of direct receptor-mediated effects remains uncertain
Immune cells	Demonstrated in selected populations, particularly intestinal intraepithelial lymphocytes; expression in conventional circulating monocytes and tissue macrophages is inconsistent [44,77]	Broader immune-cell distribution remains poorly defined; GIPR immunoreactivity has been reported in macrophages and crown-like structures within human epicardial adipose tissue [76]	Local intestinal immune regulation and possible adipose–immune signaling; systemic anti-inflammatory effects may also be mediated indirectly through metabolic or neuroimmune pathways

The table summarizes representative, best-supported receptor localizations and is not exhaustive. Receptor abundance and cellular localization vary according to species, tissue or adipose depot, physiological or disease state, and detection method. For GLP-1R, greater weight was given to studies using validated antibodies, ligand-binding methods, genetic reporter models, or transcriptomic approaches. Cell-specific human GIPR localization is generally less comprehensively characterized. The detection of receptor transcript or protein does not establish that an observed physiological or pharmacological effect is directly receptor-mediated or quantitatively important in humans. Much of the detailed neuronal and immune-cell mapping derives from experimental animal models. Abbreviations: GIPR, glucose-dependent insulinotropic polypeptide receptor; GLP-1R, glucagon-like peptide-1 receptor.

## 7. Cardiovascular Outcome Trials of Incretin-Based Therapies

Cardiovascular outcome trials provide the clinical anchor for this review: they establish cardiovascular benefit with several GLP-1RAs but do not determine the relative contributions of weight loss, metabolic improvement, and additional inflammatory or vascular mechanisms. Cardiovascular disease remains the leading cause of morbidity and mortality in patients with type 2 diabetes, and several GLP-1RAs have demonstrated cardiovascular benefit in this high-risk population [78,79,80,81]. In LEADER, liraglutide reduced 3-point major adverse cardiovascular events (MACE) and also lowered cardiovascular and all-cause mortality [79], whereas SUSTAIN-6 showed a reduction in MACE with semaglutide, largely driven by fewer nonfatal strokes, but without a significant reduction in cardiovascular death [78]. Harmony Outcomes and REWIND further supported the cardiovascular benefit of albiglutide and dulaglutide, respectively [80,81]. Importantly, early outcome trials were not uniformly positive: lixisenatide in ELIXA, conducted in patients with type 2 diabetes after recent acute coronary syndrome, met noninferiority but did not reduce the primary composite endpoint compared with placebo, and exenatide in EXSCEL similarly met noninferiority but did not demonstrate superiority for MACE [82,83]. This variability reflects differences in trial design, statistical hierarchy, molecular structure, duration of action, patient risk profile, follow-up duration, background therapy, and endpoint composition [78,79,80,81,82,83,84,85,86]. Key characteristics and outcomes of the major incretin-based cardiovascular outcome trials, including diabetes, obesity, and active-comparator studies, are summarized in Table 2.

Meta-analyses nevertheless support a clinically meaningful class-level cardiovascular effect in type 2 diabetes. A 2018 meta-analysis of the first four GLP-1RA cardiovascular outcome trials suggested a modest but significant reduction in 3-point MACE [87]. A larger 2019 meta-analysis of seven cardiovascular outcome trials reported a 12% reduction in MACE, with significant reductions in cardiovascular death, stroke, myocardial infarction, all-cause mortality, heart failure hospitalization, and kidney outcomes [20]. An updated 2021 meta-analysis including AMPLITUDE-O found a 14% reduction in MACE, 12% reduction in all-cause mortality, 11% reduction in heart failure hospitalization, and 21% reduction in a composite kidney outcome, without clear excess of severe hypoglycemia, pancreatitis, or pancreatic cancer [88]. These pooled findings suggest that the totality of evidence is stronger than any single trial, but they should not obscure between-trial differences in population, drug exposure, and statistical design [20,87,88,89].

**Table 2:** Cardiovascular outcome trials of GLP-1 receptor agonists and dual GIP/GLP-1 receptor agonists.

Drug/Class	Trial and Population	Dose/Dosing Schedule	Comparator and Trial Design	Primary Cardiovascular Outcome	Cardiovascular Death	Body-Weight Effect vs. Comparator	Key Findings and Relevance to This Review
Lixisenatide short-acting GLP-1RA [82]	ELIXA; T2D after recent ACS	10–20 μg s.c. once daily	Matching placebo; R/DB/PC; noninferiority	4-point MACE; HR 1.02 (95% CI 0.89–1.17); CV neutral	HR 0.98 (95% CI 0.78–1.22)	PA −0.7 kg	First GLP-1RA CVOT; confirmed safety but no CV efficacy signal in a high-risk post-ACS population.
Liraglutide GLP-1RA [79]	LEADER; T2D with high CV risk	Up to 1.8 mg s.c. once daily, or maximum tolerated dose	Matching placebo; R/DB/PC; noninferiority followed by superiority	3-point MACE; HR 0.87 (95% CI 0.78–0.97)	HR 0.78 (95% CI 0.66–0.93)	PA −2.3 kg	First clear GLP-1RA trial showing reduction in MACE and CV death.
Semaglutide GLP-1RA [78]	SUSTAIN-6; T2D with high CV risk	0.5 mg or 1.0 mg s.c. once weekly	Volume-matched placebo; R/DB/PC; noninferiority	3-point MACE; HR 0.74 (95% CI 0.58–0.95); noninferior (superiority nominally achieved)	HR 0.98 (95% CI 0.65–1.48)	PA −2.9 kg (0.5 mg) and −4.3 kg (1.0 mg)	Demonstrated significant MACE reduction, with the strongest effect observed for nonfatal stroke; retinopathy findings should be interpreted in the context of rapid glycemic improvement.
Exenatide extended-release GLP-1RA [83]	EXSCEL; T2D with or without previous CVD	2 mg s.c. once weekly	Matching placebo; R/DB/PC; noninferiority followed by superiority	3-point MACE; HR 0.91 (95% CI 0.83–1.00); noninferior, not superior	HR 0.88 (95% CI 0.76–1.02)	PA −1.27 kg	Confirmed cardiovascular safety but did not demonstrate superiority for MACE reduction.
Albiglutide GLP-1RA [80]	HARMONY Outcomes; T2D with established CVD	30 mg s.c. once weekly, increased to 50 mg if needed	Matching placebo; R/DB/PC; noninferiority followed by superiority	3-point MACE; HR 0.78 (95% CI 0.68–0.90)	HR 0.93 (95% CI 0.73–1.19)	PA −0.83 kg	Demonstrated MACE reduction despite modest weight change; however, the trial does not identify the mechanisms responsible for benefit.
Dulaglutide GLP-1RA [81]	REWIND; T2D with previous CVD or CV risk factors; only ~31% with established CVD	1.5 mg s.c. once weekly	Same-volume masked placebo; R/DB/PC; superiority	3-point MACE; HR 0.88 (95% CI 0.79–0.99)	HR 0.91 (95% CI 0.78–1.06)	PA −1.46 kg	Extended cardiovascular benefit to a broader and lower-risk T2D population, including many without established CVD.
Oral semaglutide GLP-1RA [84]	PIONEER 6; T2D with high CV risk	Target 14 mg orally once daily after dose escalation	Matching oral placebo; R/DB/PC; noninferiority	3-point MACE; HR 0.79 (95% CI 0.57–1.11); noninferior, not superior	HR 0.49 (95% CI 0.27–0.92)	PA −3.4 kg	Preapproval cardiovascular safety trial that suggested a potential mortality benefit.
Efpeglenatide exendin-based GLP-1RA [86]	AMPLITUDE-O; T2D with CVD and/or kidney disease	4 mg or 6 mg s.c. once weekly	Matching placebo; R/DB/PC; noninferiority followed by superiority	3-point MACE; HR 0.73 (95% CI 0.58–0.92)	HR 0.72 (95% CI 0.50–1.03)	PA −2.6 kg	Supports CV benefit across structurally diverse GLP-1RAs, including an exendin-based agent.
Oral semaglutide GLP-1RA [85]	SOUL; T2D with ASCVD and/or CKD	3 mg orally once daily, escalated to 7 mg and then 14 mg	Matching oral placebo; R/DB/PC; superiority	3-point MACE; HR 0.86 (95% CI 0.77–0.96)	HR 0.93 (95% CI 0.80–1.09)	PA −2.95 kg	Confirms CV benefit of oral semaglutide after PIONEER 6 had established CV safety.
Semaglutide 2.4 mg GLP-1RA [90]	SELECT; overweight or obesity, established CVD, without diabetes	2.4 mg s.c. once weekly after dose escalation	Matching placebo; R/DB/PC; superiority	3-point MACE; HR 0.80 (95% CI 0.72–0.90)	HR 0.85 (95% CI 0.71–1.01)	PA −8.51 percentage points	Demonstrated MACE reduction with semaglutide in secondary-prevention patients with overweight or obesity without diabetes.
Tirzepatide dual GIP/GLP-1RA [21]	SURPASS-CVOT; T2D with established ASCVD	2.5 mg s.c. once weekly, escalated by 2.5 mg every 4 weeks up to 15 mg or maximum tolerated dose	Dulaglutide 1.5 mg once weekly; R/DB/AC; noninferiority with superiority assessment	3-point MACE; HR 0.92 (95.3% CI 0.83–1.01); noninferior, not superior	HR 0.89 (95% CI 0.77–1.02)	−6.8 percentage points difference vs. dulaglutide	Tirzepatide was noninferior, but not superior, to dulaglutide for 3-point MACE. The trial therefore does not establish incremental ASCVD protection from dual GIP/GLP-1 receptor agonism despite greater weight loss and metabolic improvement.

Hazard ratios below 1.0 favor the active drug. MACE generally refers to cardiovascular death, nonfatal myocardial infarction, or nonfatal stroke, except in ELIXA, where hospitalization for unstable angina was also included. Body-weight effects are reported as presented in the original publications and are not directly comparable across trials because of differences in population, baseline weight, diabetes status, follow-up duration, dose, and comparator. In active-comparator trials, weight effects are relative to the active comparator rather than placebo. Individual components of MACE, including cardiovascular death, were often secondary or exploratory and should be interpreted cautiously. Abbreviations: AC, active-comparator-controlled; ACS, acute coronary syndrome; ASCVD, atherosclerotic cardiovascular disease; CI, confidence interval; CKD, chronic kidney disease; CV, cardiovascular; CVD, cardiovascular disease; CVOT, cardiovascular outcomes trial; DB, double-blind; GIP, glucose-dependent insulinotropic polypeptide; GLP-1RA, glucagon-like peptide-1 receptor agonist; HR, hazard ratio; MACE, major adverse cardiovascular events; PA, placebo-adjusted; PC, placebo-controlled; R, randomized; s.c., subcutaneous; T2D, type 2 diabetes.

The SELECT trial extended cardiovascular outcome evidence for GLP-1 receptor agonism to patients with overweight or obesity without diabetes. In 17,604 patients with established cardiovascular disease, body mass index (BMI) ≥ 27 kg/m^2^, and no diabetes, semaglutide 2.4 mg once weekly reduced the primary composite endpoint of cardiovascular death, nonfatal myocardial infarction, or nonfatal stroke compared with placebo (6.5% vs. 8.0%; hazard ratio [HR] 0.80, 95% confidence interval [CI] 0.72–0.90). However, discontinuation due to adverse events was more frequent with semaglutide, largely reflecting gastrointestinal tolerability [90].

In a subsequent prespecified analysis, the cardiovascular benefit of semaglutide was consistent across baseline weight and waist-circumference categories, early weight loss did not predict subsequent MACE reduction, and mediation analysis estimated that approximately 33% of the observed benefit was mediated through reduction in waist circumference [22]. These findings suggest that changes in adiposity alone may not fully account for the observed cardiovascular benefit. However, mediation analyses cannot establish specific weight-independent mechanisms, and the potential contributions of inflammation, endothelial function, thrombosis, plaque biology, and visceral or ectopic adipose tissue remain to be clarified [22,89]. Post hoc LEADER data similarly showed cardiovascular benefit across baseline LDL-C strata and statin-use groups, suggesting that GLP-1RA-mediated benefit may complement established lipid-lowering therapy [91].

For dual GIP/GLP-1 receptor agonism, the evidence is more recent and requires careful interpretation. In SURPASS-CVOT, tirzepatide was compared with dulaglutide, an established GLP-1RA, in patients with type 2 diabetes and atherosclerotic cardiovascular disease (ASCVD). Tirzepatide, titrated up to 15 mg weekly, produced greater reductions in body weight, glycated hemoglobin (HbA1c), triglycerides, and other metabolic risk factors than dulaglutide 1.5 mg weekly, but met noninferiority rather than superiority for 3-point MACE (12.2% vs. 13.1%; HR 0.92, 95.3% CI 0.83–1.01) [21]. Thus, the trial demonstrated that tirzepatide was noninferior to dulaglutide for MACE but did not establish incremental ASCVD protection attributable to dual GIP/GLP-1 receptor agonism. Although tirzepatide produced greater weight loss and metabolic improvement without a statistically superior reduction in MACE, the trial was not designed to isolate the cardiovascular contribution of GIPR activation or to determine whether weight loss mediated cardiovascular outcomes. Whether GIP receptor activation has direct anti-inflammatory or vascular effects independent of weight, glycemia, blood pressure, and lipid changes therefore remains unresolved. Additional evidence is awaited from SURMOUNT-MMO (NCT05556512), which is testing tirzepatide versus placebo in adults with overweight or obesity but without diabetes, including both primary- and secondary-prevention populations [92].

Direct comparisons between semaglutide and tirzepatide should be interpreted cautiously. In SURMOUNT-5, tirzepatide 10 or 15 mg produced greater mean weight loss than semaglutide 1.7 or 2.4 mg at 72 weeks (−20.2% vs. −13.7%) [93]. The trial did not evaluate semaglutide 7.2 mg, which subsequently produced greater weight loss than semaglutide 2.4 mg in STEP UP [94]. Because these studies differed in design and were not cardiovascular outcome trials, their weight-loss findings should not be used to infer comparative cardiovascular efficacy. In a real-world study of patients with type 2 diabetes, no clear difference in myocardial infarction, stroke, or all-cause mortality was observed between tirzepatide and semaglutide [95]. Dedicated head-to-head outcome trials are needed to determine whether differences in weight loss translate into differences in cardiovascular risk reduction.

## 8. Translational and Human Evidence for Vascular and Anti-Atherosclerotic Effects

Despite strong experimental evidence, human vascular imaging studies have not consistently demonstrated reductions in arterial inflammation or plaque burden with GLP-1RAs. In LIRAFLAME, liraglutide did not significantly reduce arterial inflammation assessed by fluorine-18 fluorodeoxyglucose ([^18^F]FDG) positron emission tomography/computed tomography (PET/CT) in patients with type 2 diabetes, although exploratory analyses suggested a possible signal in those with established cardiovascular disease [96]. A smaller LIRAFLAME substudy using copper-64-labeled DOTA-Tyr^3^-octreotate ([^64^Cu]Cu-DOTATATE), a macrophage-targeted tracer, showed reductions in coronary uptake within the liraglutide group, but between-group differences versus placebo were only borderline, making the findings hypothesis-generating rather than definitive [97]. More recently, a randomized positron emission tomography-magnetic resonance imaging (PET-MRI) trial of semaglutide 1.0 mg in patients with type 2 diabetes and cardiovascular disease found no significant reduction in carotid plaque inflammation using either [^18^F]FDG or gallium-68-labeled DOTA-Tyr^3^-octreotate ([^68^Ga]DOTATATE), likely reflecting low baseline inflammatory activity and a well-treated trial population [98].

Plaque-structure studies convey similarly inconclusive evidence. In an 18-month randomized trial, once-weekly exenatide improved glycemic control but did not reduce carotid plaque volume, plaque composition, or endothelial function compared with placebo [99,100]. Conversely, non-randomized data with liraglutide suggested improvement in carotid intima-media thickness, but such findings remain vulnerable to confounding [101]. The STOP trial was designed to assess whether semaglutide slows coronary plaque progression as determined by serial coronary computed tomography angiography (CCTA), with non-calcified plaque volume as the primary endpoint; however, peer-reviewed primary plaque results have not yet been published [102]. In a recent prospective cohort of patients after percutaneous coronary intervention (PCI) with type 2 diabetes, GLP-1RA use was associated with lower MACE, in-stent restenosis, and non-target lesion progression after propensity-score matching, but these observational findings require confirmation in randomized plaque-imaging studies [103]. Thus, current human imaging data do not yet provide a simple explanation for the event reduction seen in outcome trials. Rather, they suggest that vascular benefit may depend on patient selection, baseline inflammatory burden, treatment duration, imaging modality, and whether the endpoint captures inflammation, plaque volume, or plaque stability.

Epicardial adipose tissue may represent a particularly relevant link between obesity, inflammation, and coronary disease [104]. Unlike many other adipose depots, epicardial fat lies in direct contiguity with the myocardium and coronary arteries, without a fascial barrier, allowing local paracrine and inflammatory crosstalk. Human epicardial adipose tissue expresses receptors for GLP-1, GIP, and glucagon, with GIP and glucagon receptor protein expression localized particularly in macrophages and crown-like structures, supporting the concept that incretin-based therapies could modulate this depot directly [75,76]. In a prespecified STOP analysis, semaglutide reduced epicardial adipose tissue volume by approximately 9%, but also increased computed tomography (CT) attenuation/density, a finding that requires cautious interpretation because higher attenuation may reflect inflammation, fibrosis, adipocyte remodeling, or a dynamic tissue response rather than a uniformly beneficial change [105,106].

For dual GIP/GLP-1 receptor agonism, direct vascular evidence remains limited. The ongoing T-PLAQUE trial (NCT05708859) is designed to assess whether tirzepatide slows coronary atherosclerosis progression using multidetector CT angiography in patients with type 2 diabetes and coronary plaque, with change in non-calcified plaque volume as the primary endpoint [107]. Until such data are available, the anti-atherosclerotic interpretation of tirzepatide remains based mainly on its strong effects on weight, glycemia, blood pressure, lipids, and systemic inflammation, rather than direct evidence of plaque modification.

## 9. Future Directions

Future studies should quantify the relative contributions of weight loss, metabolic improvement, vascular inflammation, ectopic adipose-tissue remodeling, and plaque biology to the cardiovascular effects of incretin-based therapies. For tirzepatide, the key pending evidence is SURMOUNT-MMO (NCT05556512), an event-driven trial in adults with overweight or obesity but without diabetes, including both primary- and secondary-prevention populations [92].

The expanding incretin pipeline will test whether next-generation incretin combinations improve cardiovascular outcomes; greater weight loss should not be assumed to confer greater ASCVD protection. Retatrutide, a triple GIP/GLP-1/glucagon receptor agonist, and survodutide, a GLP-1/glucagon receptor dual agonist, have produced substantial weight loss in phase 2 obesity trials, while CagriSema combines semaglutide with the amylin analog cagrilintide [108,109,110]. Dedicated cardiovascular outcome trials are now extending beyond semaglutide and tirzepatide, including REDEFINE 3 with CagriSema (NCT05669755), SYNCHRONIZE-CVOT with survodutide (NCT06077864), and a retatrutide cardiovascular outcomes trial (NCT06383390) [111].

Mechanistic studies should move beyond BMI and total body weight. Prespecified mediation analyses should integrate waist circumference, visceral and hepatic fat, epicardial and perivascular adipose tissue, glycemia, blood pressure, lipids, hsCRP, IL-6, and other inflammatory biomarkers. Imaging approaches such as coronary fat attenuation index may be particularly useful, because they can non-invasively assess perivascular inflammation on CCTA and may help distinguish plaque regression from plaque stabilization or reduced inflammatory activity [112,113].

Finally, future trials should define which patients derive the greatest vascular benefit. Patients with visceral obesity, residual inflammatory risk, elevated hsCRP or IL-6, high-risk plaque features, or increased perivascular inflammatory imaging signals may represent the most informative populations. Combination strategies with lipid-lowering or dedicated anti-inflammatory therapies are also attractive, but should be tested cautiously, with attention to infection risk, tolerability, and hard cardiovascular endpoints rather than biomarker changes alone.

## 10. Limitations

Several limitations should be acknowledged. This is a narrative rather than systematic review, and no formal meta-analysis was performed. Evidence on direct vascular and anti-inflammatory effects of incretin-based therapies remains heterogeneous, with many data derived from preclinical models, biomarker analyses, or small imaging studies. In addition, comparisons between semaglutide, tirzepatide, and emerging multi-agonists are limited by differences in study populations, doses, comparators, follow-up duration, and endpoints.

## 11. Conclusions

Obesity amplifies atherosclerotic risk through adipose-tissue dysfunction, chronic low-grade inflammation, and adverse effects on the vascular wall. Incretin-based therapies, particularly GLP-1RAs, reduce cardiovascular events and improve metabolic and inflammatory biomarkers, but direct human evidence for receptor-mediated anti-atherosclerotic effects remains limited. The current evidence therefore supports a combined model in which weight loss and metabolic improvement likely contribute substantially, while additional inflammatory, neuroimmune, endothelial, and plaque-related mechanisms remain biologically plausible but incompletely proven.

## Data Availability

No new data were created or analyzed in this study. Data sharing is not applicable to this article.
